# A Pharmacovigilance Study Regarding the Risk of Antibiotic-Associated *Clostridioides difficile* Infection Based on Reports from the EudraVigilance Database: Analysis of Some of the Most Used Antibiotics in Intensive Care Units

**DOI:** 10.3390/ph16111585

**Published:** 2023-11-09

**Authors:** Bogdan Ioan Vintila, Anca Maria Arseniu, Claudiu Morgovan, Anca Butuca, Mihai Sava, Victoria Bîrluțiu, Luca Liviu Rus, Steliana Ghibu, Alina Simona Bereanu, Ioana Roxana Codru, Felicia Gabriela Gligor

**Affiliations:** 1 Clinical Surgical Department, Faculty of Medicine, “Lucian Blaga” University of Sibiu, 550169 Sibiu, Romania; bogdan.vintila@ulbsibiu.ro (B.I.V.); alina.bereanu@ulbsibiu.ro (A.S.B.); ioanaroxana.bera@ulbsibiu.ro (I.R.C.); 2 County Clinical Emergency Hospital, 550245 Sibiu, Romania; victoria.birlutiu@ulbsibiu.ro; 3 Preclinical Department, Faculty of Medicine, “Lucian Blaga” University of Sibiu, 550169 Sibiu, Romania; claudiu.morgovan@ulbsibiu.ro (C.M.); anca.butuca@ulbsibiu.ro (A.B.); liviu.rus@ulbsibiu.ro (L.L.R.); felicia.gligor@ulbsibiu.ro (F.G.G.); 4 Clinical Medical Department, Faculty of Medicine, “Lucian Blaga” University of Sibiu, 550169 Sibiu, Romania; 5 Department of Pharmacology, Physiology and Pathophysiology, Faculty of Pharmacy, “Iuliu Hatieganu” University of Medicine and Pharmacy, 400012 Cluj-Napoca, Romania; steliana.ghibu@umfcluj.ro

**Keywords:** *Clostridioides difficile* infection, antibiotic, EudraVigilance, descriptive analysis, disproportionality analysis, pharmacovigilance

## Abstract

The Gram-positive anaerobic bacterium *Clostridioides difficile* (CD) can produce intense exotoxins, contributing to nosocomial infections, and it is the most common cause of health-care-associated infectious diarrhea. Based on spontaneous Individual Case Safety Reports from EudraVigilance (EV), we conducted a descriptive analysis of *Clostridioides difficile* infection (CDI) cases that reported a spontaneous adverse reaction related to using ceftriaxone, colistimethate, ciprofloxacin, gentamicin, linezolid, meropenem, and piperacillin/tazobactam. Most ADR reports registered in EV that were related to CDI were associated with ceftriaxone (33%), ciprofloxacin (28%), and piperacillin/tazobactam (21%). Additionally, the disproportionality analysis performed showed that all studied antibiotics had a lower reporting probability when compared to clindamycin. A causal relationship between a drug and the occurrence of an adverse reaction cannot be established from EV data alone because the phenomena of underreporting, overreporting, and reporting bias may affect the results. Based on the analysis of the collected data, this study underlines the importance of surveillance and monitoring programs for the consumption of antibiotics. Furthermore, it is essential to use standardized laboratory tests to define CDI’s nature accurately. To prevent this infection, specialists should collaborate and adhere strictly to antibiotic stewardship programs, hygiene practices, and isolation protocols.

## 1. Introduction

*Clostridioides difficile* (CD) was first identified in 1935 [1], and after all this time, it remains the most common cause of health-care-associated infectious diarrhea [2]; it is associated with an impressive number of infections, around 152,905 cases in Europe, resulting in 8382 deaths annually [3]. 

Patients requiring admission to the intensive care unit are at increased risk of developing *Clostridioides difficile* infection (CDI) [3]. The reason may be the severe comorbidities, the invasive procedures performed in this setting, and the extended use of broad-spectrum antibiotics (third-generation cephalosporins, carbapenems, etc.) [4,5]. 

There is a heterogeneous relationship between antibiotic use in the intensive care unit (ICU) and CDI. The most used antibiotics in the ICU are non-selective in their action, disrupting gut microbiota and creating an environment in which *C. difficile* thrives and produces toxins that cause CDI-associated diarrhea [6]. Through understanding the unique characteristics of intensive care patients, healthcare providers can provide them with personalized treatment, allowing them to administer antibiotics at the appropriate dose. As a result of this personalized approach to treatment, adverse events can be minimized, outcomes can be improved, and cost-effectiveness can be reached [7].

Roughly 71% of patients admitted to the ICU receive antibiotic treatment, significantly preserving millions of lives [8,9]. Due to their broad-spectrum activity and effectiveness against various pathogens, meropenem (MER), linezolid (LIN), and colistin (COL) are commonly used to treat infections produced by multidrug-resistant microorganisms found in ICU patients [10,11,12,13,14].

The carbapenem family of antibacterials is well known for its broad range of effectiveness, making them the most frequently prescribed antibiotics in intensive care units for drug-resistant infections [15,16]. A recent study found that prior exposure to MER was present in 27.60% of patients with CDI, preceded only by piperacillin/tazobactam (PIP/TAZ) in 77.60% of cases of CDI [17].

LIN belongs to the class of oxazolidinone antibiotics and is authorized to treat infections caused by Gram-positive pathogens [11,18]. Diarrhea is one of the most frequently encountered adverse effects of linezolid—but, in this case, factors contributing to CD colitis require further discussion. Hence, LIN is considered to have a low risk of CDI [19].

Polymyxins, comprising polymyxin B and polymyxin E (colistin or colistimethate), are a category of antibiotics introduced into clinical practice in the 1950s [20]. The emergence of multidrug-resistant Gram-negative bacteria has reaffirmed the significance of COL in clinical settings. This molecule is beneficial, particularly in the intensive care department, where many bacteria remain unresponsive to other antibacterial medications currently available for clinical use [20]. COL treatment was identified as an independent risk factor for 30-day mortality in CDI [21]. Still, we must consider that patients receiving colistin already have a critical health condition and an increased risk of mortality and morbidity.

Other antibiotics can be administered in an intensive care unit, including PIP/TAZ, ceftriaxone (CFT), ciprofloxacin (CPX), and gentamycin (GEN). PIP/TAZ is a potent β-lactam/β-lactamase inhibitor antibiotic commonly prescribed in the intensive care unit [22,23]. A retrospective review of medical records of 640 patients in an ICU found that 73.4% of patients received CFT. Less than 3% of patients in ICUs receive CPX and GEN [24]. An advantage of CPX and GEN in treating critically ill patients is that they can be used in pathogens less susceptible to usual antibiotics used in intensive care, especially for urinary tract infections [25,26].

The therapeutic strategy in managing CDI involves an intricated approach that includes triggers discontinuation (e.g., antibiotics, proton pump inhibitors, etc.). Patients must be isolated and stabilized, and a personalized antibiotic regimen based on severity must be prescribed, along with interventional endoscopy or surgery if other measures have failed [27,28].

Currently, fecal microbiota transplantation is only approved for recurrent or refractory CDI in adults, as recommended by the updated treatment guidance document on CDI in adults, according to the European Society of Clinical Microbiology and Infectious Diseases [29]. A retrospective study by Popa et al. concluded that fecal microbiota transplantation has a meager recurrence rate in the primary severe CDI [30]. Also, patients with CDI and COVID-19 may benefit from fecal microbiota transplantation as an effective method of ensuring and optimizing safety and efficacy [31]. 

Gastrointestinal infections caused by this anaerobic bacterium have resulted in prolonged hospitalizations, escalating healthcare costs, and even life-threatening complications for vulnerable populations of patients. It is a prominent and pressing concern in microbiology and healthcare. 

The incidence of adverse events associated with the use of antibiotics in clinical settings is significant due to the common prescription of this drug class. It is well known that prescribing broad-spectrum antibiotics can lead to CDI, one of the most prevalent adverse reactions. In this study, we reviewed CDI cases spontaneously reported as adverse drug reactions associated with the use of ceftriaxone, colistimethate, ciprofloxacin, gentamicin, linezolid, meropenem, and piperacillin/tazobactam using data submitted to EudraVigilance. The choice of antibiotics for this study is based on various factors, including clinical relevance, mechanism, and spectrum of activity. Fluoroquinolones, cephalosporins, carbapenems, aminoglycosides, and antibiotics with broad-spectrum activity, such as piperacillin/tazobactam, may induce CDI [24,32,33,34,35]. Colistin is becoming a valuable therapeutic alternative for infections caused by some multidrug-resistant Gram-negative pathogens [36]. COL and LIN have a complementary spectrum and are used simultaneously with other broad-spectrum antimicrobials among the antibiotics; consequently, the risk for CDI is more significant with this association [37]. Clindamycin, an antibiotic with a great risk for CDI, was not included in our analysis because it is used rarely in ICU, like in pulmonary infection with anaerobic organisms [23].

## 2. Results

### 2.1. Descriptive Analysis of Reports during the 1 January 2003 and 7 August 2023 Period

#### 2.1.1. Total Number of Reports Related to *Clostridioides difficile* Infection

(a) Total ICSRs

**Figure 1:**
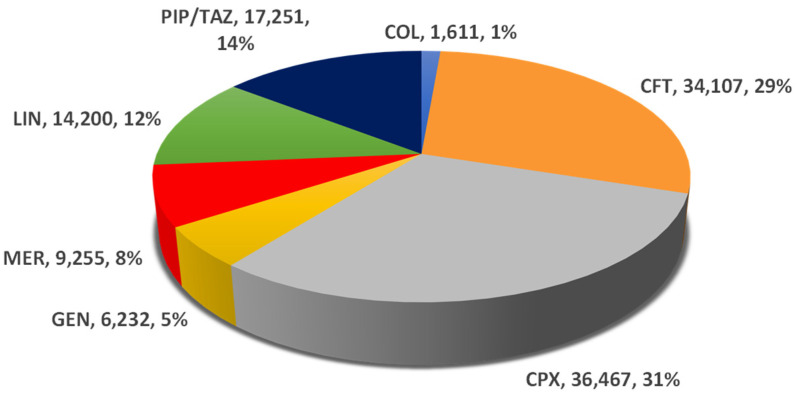
Total ADRs related to *Clostridioides difficile* infection reported in EV. CFT—ceftriaxone; COL—colistimethate; CPX—ciprofloxacin; GEN—gentamicin; LIN—linezolid; MER—meropenem; PIP/TAZ—piperacillin and tazobactam.

From the total number of 119,123 adverse drug reactions (ADRs) reported in EV until 7 August 2023 for the seven antibiotics (CFT, CPX, COL, GEN, LIN, MER, and PIP/TAZ), the higher proportion was calculated for CPX (31%) and the lower ratio for COL (1%). Also, a higher proportion was observed for CFT (29%), PIP/TAZ (14%), and LIN (12%). MER and GEN have registered only 8% and 5%, respectively, of the total ADRs related to CDI. (Figure 1).

(b) Total ADRs related to *Clostridioides difficile*

**Figure 2:**
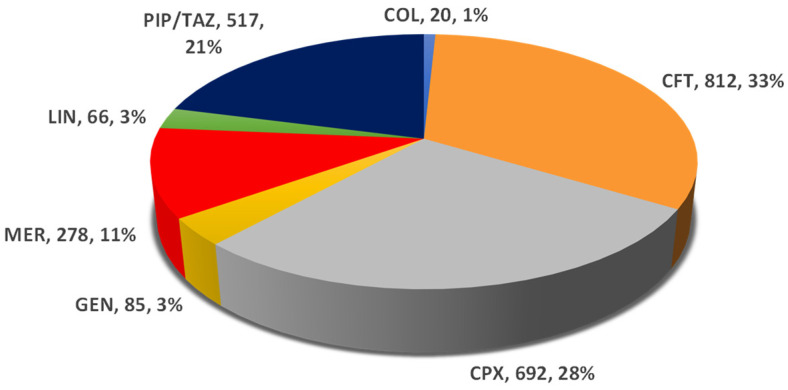
Total ADRs related to *Clostridioides difficile* infection. CFT—ceftriaxone; COL—colistimethate; CPX—ciprofloxacin; GEN—gentamicin; LIN—linezolid; MER—meropenem; PIP/TAZ—piperacillin and tazobactam.

Regarding the CDI, the higher proportions from the total value reported for the seven drugs were registered for CFT (33%), CPX (28%), and PIP/TAZ (21%). It is noticed that MER has 11%, and the other drugs have a value under 3% (GEN—3%, LIN—3%, and COL—1%) (Figure 2).

(c) Proportion of ADRs related to *Clostridioides difficile* from total ICSRs

**Figure 3:**
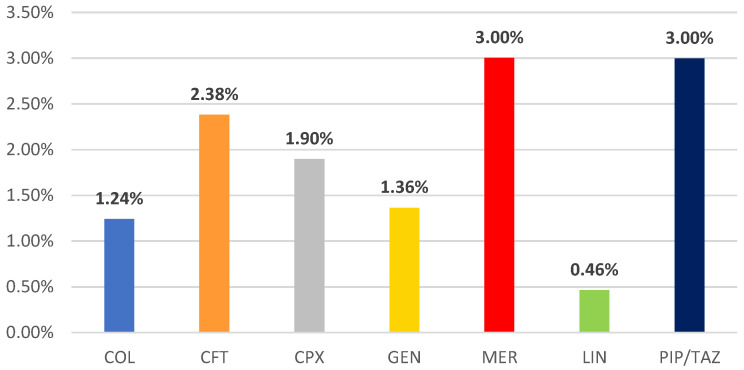
Proportion of CDI reports from total ICSRs for each antibiotic. CFT—ceftriaxone; COL—colistimethate; CPX—ciprofloxacin; GEN—gentamicin; LIN—linezolid; MER—meropenem; PIP/TAZ—piperacillin and tazobactam.

Figure 3 presents the proportion of CDI reports from total ICSRs for each antibiotic. Even though the number of reports for CDI was highest for CFT, it could be noticed that its proportion of ADRs related to CDI from the total ICSRs is situated in second place (2.38%) after MER (3%) and PIP/TAZ (3%). Values higher than 1% were calculated for CPX (1.90%), GEN (1.36%), and COL (1.24%). Only LIN (0.46%) has a smaller value than 1%.

#### 2.1.2. Evolution of ICSR Number Related to *Clostridioides difficile* Infection

**Figure 4:**
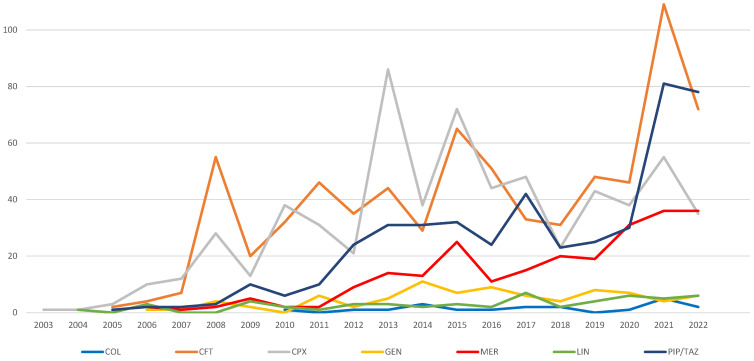
Evolution of reports related to *Clostridioides difficile* infection in EV between 1 January 2003 and 31 December 2022. CFT—ceftriaxone; COL—colistimethate; CPX—ciprofloxacin; GEN—gentamicin; LIN—linezolid; MER—meropenem; PIP/TAZ—piperacillin and tazobactam.

During the 2003–2022 period, an ascendant evolution of reported ADRs related to CDI could be observed in all series (Figure 4). Analyzing the chronological series, it can be observed that the highest number of reports was registered for CPX (in 2007, 2010, 2013, 2014, 2015, and 2017), CFT (in 2008, 2009, 2011, 2012, 2016, 2018, 2019, 2020, and 2021), and PIP/TAZ (in 2022) (Figure 4). 

The retrospective analysis of ICSRs reported in EV between 1 January 2003 and 31 December 2022 shows the highest average reports per year for CFT (40.5, 95% CI: 27.6–53.4) and PIP/TAZ (25.3, 95% CI: 13.6–36.9), and the lowest for LIN (2.8, 95% CI: 1.8–3.8) and COL (1.5, 95% CI: 0.7–2.3). For MER and GEN, the average number of reports per year was 13.6 (95% CI: 7.6–19.5) and 4.9 (95% CI: 3.3–6.5), respectively (Table 1).

#### 2.1.3. Distribution of CDI-Related Reports by Outcome

**Figure 5:**
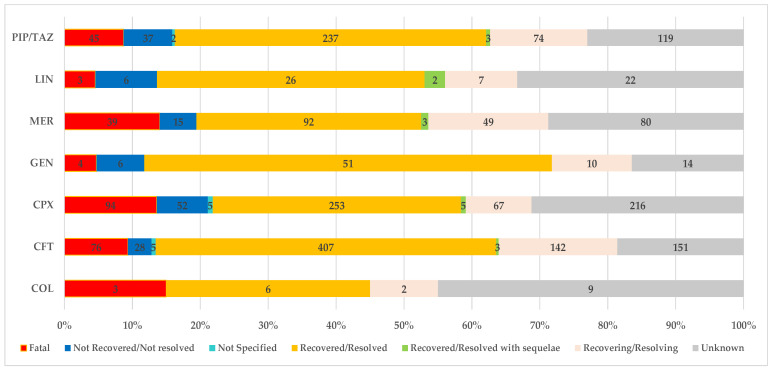
Distribution of CDI-related reports by outcome. CFT—ceftriaxone; COL—colistimethate; CPX—ciprofloxacin; GEN—gentamicin; LIN—linezolid; MER—meropenem; PIP/TAZ—piperacillin and tazobactam.

From the total reports related to CDI, the highest frequency of an unfavorable outcome (fatal, not recovered/not resolved, or recovered/resolved with sequelae) was registered for CPX (21.8%) and MER (20.5%). The lowest frequency of an unfavorable outcome of CDI-related ADRs was calculated for GEN (11.8%) and CFT (13.2%). Similar proportions were registered for COL (15%), LIN (16.7%), and PIP/TAZ (16.8%). Thus, the highest frequency of fatal ADRs was for COL (15%), MER (14%), and CPX (13.6%). Values less than 10% were noticed for CFT (9.4%), PIP/TAZ (8.7%), GEN (4.7%), and LIN (4.5%). Regarding the CDI reported as not recovered/not resolved, the proportions registered were as follows: 9.1% (LIN), 7.5% (CPX), 7.2% (PIP/TAZ), 7.1% (GEN), 5.4% (MER), and 3.4% (CFT). No ADR was reported for COL. The presence of sequelae was noticed in 3% of cases declared recovered or resolved associated with LIN, 1.1% with MER, 0.7% with CPX, 0.6% with PIP/TAZ, 0.4% with CFT. Also, no reports were registered for COL and GEN (Figure 5).

### 2.2. Disproportionality Analysis for Data Reported between 1 January 2003 and 7 August 2023

**Figure 6:**
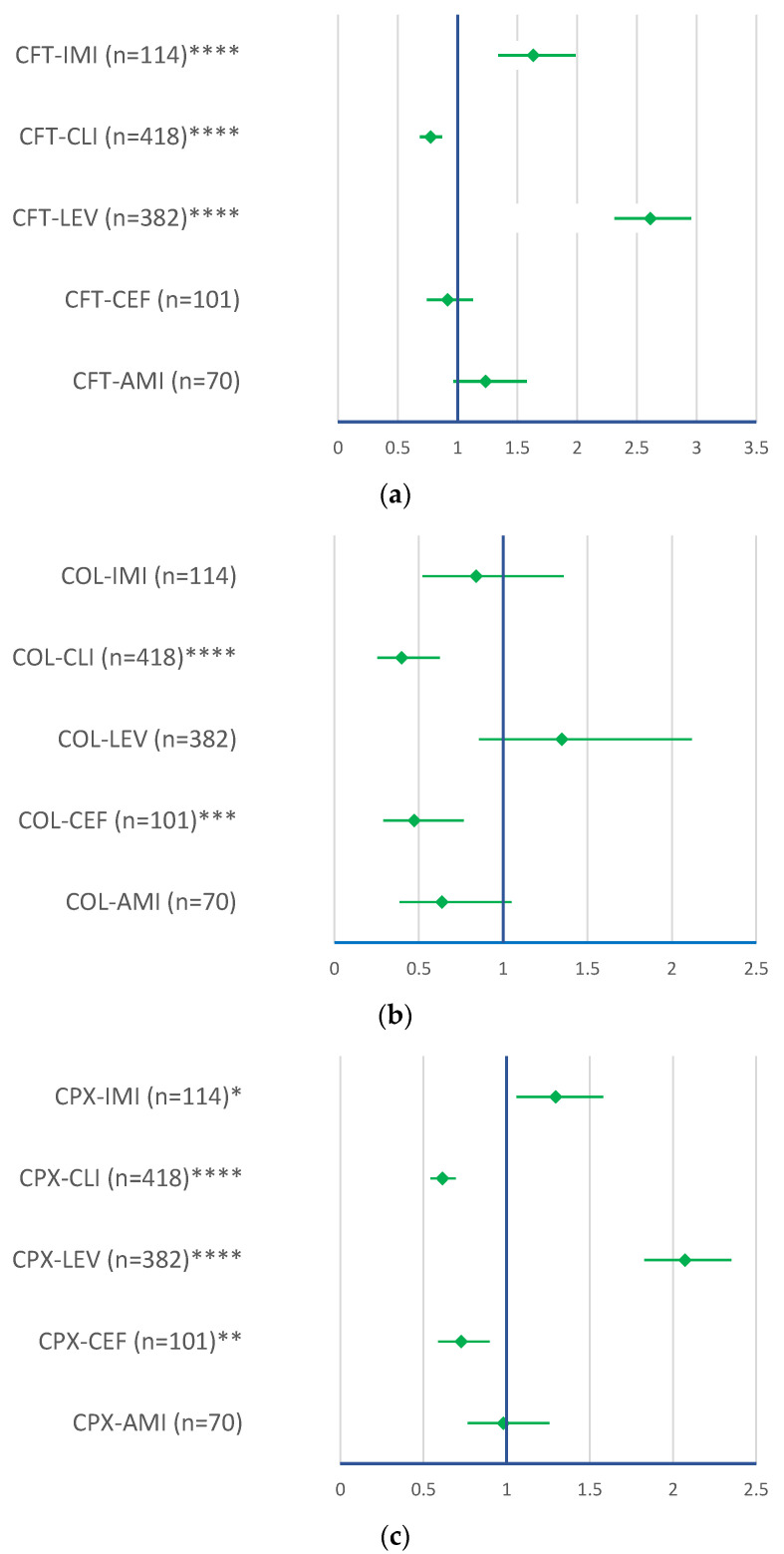

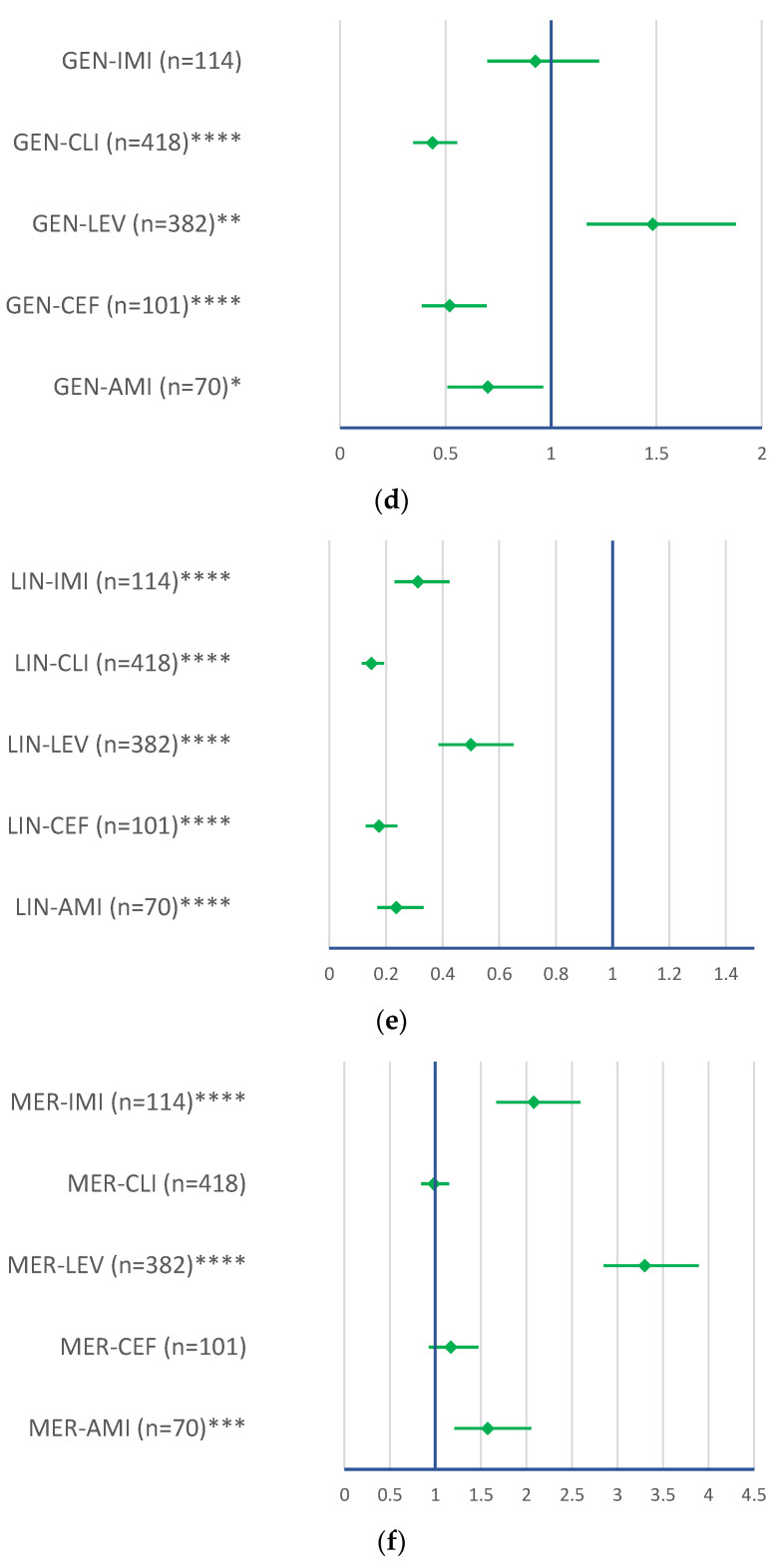

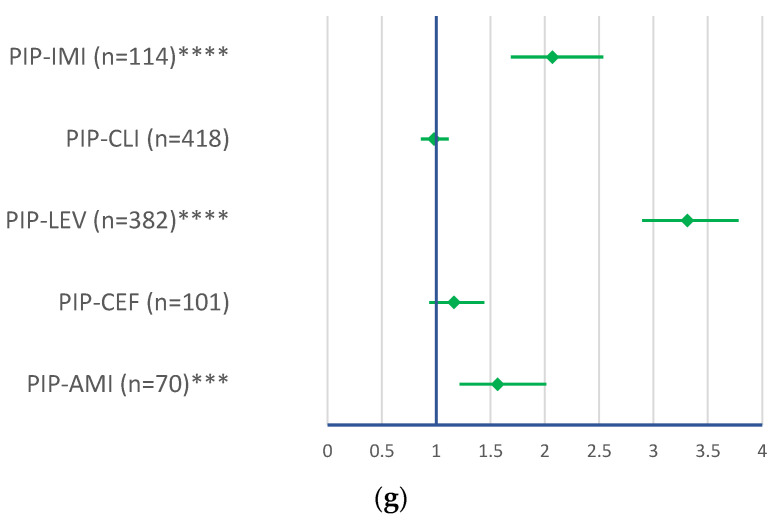
Disproportionality analysis for ADRs related to *Clostridioides difficile* infection. (**a**) Ceftriaxone; (**b**) colistimethate; (**c**) ciprofloxacin; (**d**) gentamicin; (**e**) linezolid; (**f**) meropenem; (**g**) piperacillin and tazobactam. AMI—amikacin; CEF—ceftazidime; CLI—clindamycin; CFT—ceftriaxone; COL—colistimethate; CPX—ciprofloxacin; GEN—gentamicin; IMI—imipenem/cilastatin; LEV—levofloxacin; LIN—linezolid; MER—meropenem; PIP/TAZ—piperacillin and tazobactam. * *p* <0.05; ** *p* ≤ 0.01; *** *p* ≤ 0.001; **** *p* ≤ 0.0001.

The disproportionality analysis was realized through a comparison with amikacin (AMI), ceftazidime (CEF), clindamycin (CLI), imipenem/cilastatin (IMI), and levofloxacin (LEV). A higher probability of CDI reporting for CFT compared to LEV (ROR: 2.6136 ± 0.3011) and IMI (ROR: 1.6334 ± 0.2929) and for GEN compared to LEV (ROR: 1.4819 ± 0.3122) was observed. CDI cases related to CPX have a higher probability of reporting than LEV (ROR: 2.073 ± 0.245) and IMI (ROR: 1.2955 ± 0.2345). It is more probable that MER and PIP/TAZ have a higher frequency of reporting compared to AMI (ROR: 1.574 ± 0.3664 for MER and ROR: 1.5651 ± 0.349—for PIP/TAZ), LEV (ROR: 3.299 ± 0.4506—MER and ROR: 3.311 ± 0.4137—PIP/TAZ), and IMI (ROR: 2.081 ± 0.4111—MER and ROR: 2.0692 ± 0.3829). Colistin does not have a higher probability of reporting than each of the drug chosen for reference. A lower statistical probability of CDI reporting (*p* < 0.05) was observed for (i) CFT versus CLI; (ii) COL versus CEF and CLI; (iii) CPX versus CEF and CLI; (iv) GEN versus AMI, CEF, CLI; and (v) LIN versus all drugs (AMI, CEF, LEV, CLI, and IMI) (Figure 6).

#### Forecasting the Number of ICSRs Related to Clostridioides Difficile Infection for 2023–2025 for the Analyzed Antibiotics

The forecast of the CDI cases related to the analyzed drugs was predicted based on chronological series. Yearly, an increasing in reports with 0.15 (COL), 0.3 (GEN and LIN), 2.1 (MER), 2.5 (CPX), 3.5 (CFT), and 3.7 (PIP/TAZ) was observed (Table 2). The model proposed showed a significant upward trend for all antibiotics (*p* < 0.05), except COL (*p* > 0.05) (Table 2). 

## 3. Discussion

CDI is considered a severe and potentially life-threatening side effect of antibiotics, with an overall incidence rate of 101.3 cases per 100,000 people in the United States in 2020 and an associated healthcare cost of USD 6.3 billion. Moreover, according to a report by the European Centre for Disease Prevention and Control, the crude incidence density of CDI during 2016–2017 was 3.48 cases per 10,000 patient days [38].

Many risk factors have been identified as potentially increasing the risk of CDI among patients recently exposed to antibiotics, with age over 65 years and prolonged hospitalization being some of them [39,40]. Thus, the risk of antibiotic exposure depends on the drug and administration route [41]. Knowing the antibiotics associated with the most significant risks of developing this type of infection is crucial to preventing CDI and improving patient outcomes.

This study aimed to investigate the risk for CDI associated with the use of seven antibiotics commonly administered to ICU patients through analyzing suspected adverse drug reaction reports submitted to EudraVigilance (EV). The total number of reports registered in EV for the seven studied antibiotics up to 7 August 2023 was 119,123, of which 31% were associated with ciprofloxacin (n = 36,467) and 29% with ceftriaxone (n = 34,107). 

Also, among the studied antibiotics, the majority of ADR reports registered in EV that were related to CDI were associated with ceftriaxone (33%), ciprofloxacin (28%), and piperacillin/tazobactam (21%). However, the percentage of the CDI-related ADRs of the total number of ICSRs registered for the seven antibiotics was 3% for meropenem and piperacillin/tazobactam, respectively, and 2.38% for ceftriaxone. A study conducted by Rafey et al. found that 93% of patients who were diagnosed with CDI received antibiotic treatment with a duration of at least four days, piperacillin/tazobactam, meropenem, ciprofloxacin, and ceftriaxone being among the most common antibiotics associated with CDI [17]. Another study analyzing the association between CDI and various antibiotics, based on reports submitted to the Food and Drug Administration Adverse Event Reporting System (FAERS), identified that lincosamides possess the highest risk for CDI, followed by monobactams, penicillin combinations, carbapenems, and cephalosporins [42]. According to Mullish and Williams, cephalosporins, fluoroquinolones, clindamycin, and certain penicillins increase the risk of developing CDI the most [43]. A meta-analysis by Vardakas et al. concluded that carbapenems were associated with more CDIs than cephalosporins and fluoroquinolones [33]. A retrospective cohort study by Pépin et al. highlighted that fluoroquinolones were most strongly associated with CDI. At the same time, third-generation cephalosporins, intravenous beta-lactam/beta-lactamase inhibitors, and other antibiotics, such as clindamycin or macrolides, were considered with intermediate risk [44]. Another retrospective cohort study performed by Althaqafi et al. found that 74.30% of patients diagnosed with CDI had previously used antibiotics, and in most cases, the antibiotics were administered intravenously (92.10%). Piperacillin-tazobactam was used by 38.8% of patients, being the most frequently used, followed by meropenem (24.29%) [45]. In a meta-analysis by Brown et al., the association between various antibiotic classes and the risk of community-associated CDI was evaluated. The most significant risk of CDI may be encountered with broad-spectrum antimicrobials or those active against Gram-negative or anaerobic bacteria, including cephalosporins, fluoroquinolones, and clindamycin. At the same time, macrolides, penicillins, tetracyclines, sulfonamides and trimethoprim have a lower risk of CDI [46].

From the distribution of CDI-related ICSRs over the years, a higher incidence of CDI-related ADRs associated with ceftriaxone (with an average of 40.5 reports per year), ciprofloxacin (an average of 32 reports per year), piperacillin/tazobactam (an average of 25.3 reports per year), and meropenem (an average of 13.6 reports per year) was observed. A retrospective epidemiological study of CDI conducted in a hospital in Romania for eight years showed an increasing trend in the frequency of CDI from 2011 to 2016, followed by a slight decrease. Moreover, 43% of CDI cases were associated with cephalosporins, followed by fluoroquinolones in 22% of patients [47].

Even though there was a higher incidence of CDI was reported for CFT (2.38% of the total ADRs), severe ADRs have a lower frequency than other antibiotics. From this perspective, CFT appears to be safer than other studied antibiotics, except GEN.

Regarding the disproportionality analysis, it was observed that all studied antibiotics showed a lower reporting probability when compared to clindamycin, which is similar to other retrospective studies based on FAERS reports [48].

Our study shows an upward trend in the number of ICSRs in the 2023–2025 period. The average case number was estimated with 95% probability. The number of cases reported for CFT is expected to be 73.8 (95% CI: 54.5–93.2) in 2023 and 80.9 (95% CI: 58.3–103.4) in 2025; for CPX, 58.0 (95% CI:40.4–75.6) in 2023 and 62.9 (95% CI: 42.7—83.1) in 2025; and for PIP/TAZ, 60.2 (95% CI: 29.2–35.5) in 2023 and 67.6 (95% CI: 73.9–83.6) in 2025 (Table 2).

## 4. Materials and Methods

### 4.1. Study Design

A retrospective pharmacovigilance study referring to CDI was performed, including a descriptive analysis based on the spontaneous reports registered in the EV database until 7 August 2023 (https://www.adrreports.eu/) [49]. Subsequently, a disproportionality analysis was performed to evaluate the probability of reporting of CDI related to the following antibiotics: piperacillin and tazobactam (PIP/TAZ), meropenem (MER), ceftriaxone (CFT), ciprofloxacin (CPX), gentamicin (GEN), colistimethate (COL), and linezolid (LIN) comparing to other molecules. No personal information regarding patients is included in the Individual Case Safety Report (ICSR). Thus, no ethics committee approval is required [50]. The ICSRs refer to the European Economic Area (EEA) or non-EEA and could be reported by healthcare professionals or non-healthcare professionals [51]. 

### 4.2. Materials

Seven antibiotics that are frequently used in intensive care were chosen: CFT, COL, CPX, GEN, LIN, MER, and PIP/TAZ. Data reported in EV was uploaded until 7 August 2023. Preferred terms (PTs) related to CDI were as follows: “Clostridial infection”, “Clostridial sepsis”, “Clostridium bacteremia”, “Clostridium colitis”, “Clostridium difficile colitis”, “Clostridium difficile infection”, and “Gastroenteritis clostridial”.

### 4.3. Data Analysis

A descriptive analysis of CDI cases that reported a spontaneous adverse reaction related to using CFT, COL, CPX, GEN, LIN, MER, and PIP/TAZ was performed. First, total ADRs and total cases of CDI for these seven drugs were centralized. Based on this data, the proportion of ADRs related to CDI from total ADRs reported was calculated. The evolution of CDI was represented using the total annual reports (2003–2022). 

Subsequently, the reports with an unfavorable outcome related to CDI were identified: (i) “Fatal”, (ii) “Not recovered or Not resolved”, and (iii) “Recovered with sequelae or Resolved with sequelae”. The percentage of the reports with unfavorable outcomes from the total of reports related to CDI was calculated.

To evaluate the probability of CDI reporting for the seven antibiotics, other antibiotics were chosen for comparison: amikacin (AMI), ceftazidime (CEF), levofloxacin (LEV), clindamycin (CLI), and imipenem/cilastatin (IMI). EMA recommends using the ROR and 95% CI for performing the disproportionality analysis [14,52,53]:

$$ROR=\frac{a\times d}{b\times c}$$

where:

*ROR* = reporting odds ratio;
*a* = evaluated ADR for targeted drug;
*b* = other ADRs for targeted drug;
*c* = evaluated ADR for the drug used for comparison;
*d* = other ADRs for the drug used for comparison.

95% CI = exp (ln (*ROR*) − 1.96 × SE{ln(*ROR*)}) to exp (ln(*ROR*) + 1.96 × SE{ln(*ROR*)})

where:

CI = confidence interval;
SE = standard error.

$$SE\{ln\left(ROR\right)\}=\sqrt{\frac{1}{a}+\frac{1}{b}+\frac{1}{c}+\frac{1}{d}}$$

For assuming disproportionate reporting, the total number of cases must be a minimum of 5 and the 95% CI of ROR must be higher than 1.0. [52].

The chronological series (2003–2022) were used to estimate the probability of reporting for 2023–2025. A simple linear regression test (α = 0.05) was used to forecast the CDI related to all seven antibiotics. The average predicted values and 95% CI were calculated. Because 2023 has not ended, the reports from this year were not included in the analysis.

## 5. Study Limitations

A causal relationship between a drug and the occurrence of an adverse reaction cannot be established from EV data alone, as they comprise spontaneous and voluntary reporting, and the lack of general data on drug use and underreporting can lead to significant bias. In addition, EV reports do not represent all available information regarding the benefit–risk balance. The number of adverse reactions should not be the basis for determining the probability of an adverse reaction, and the results should be interpreted with caution in the given context of data limitations, such as the number of people receiving the drug and the length of time since placing the drug on the market. Each case in EudraVigilance generally refers to a single patient; however, several adverse reactions may be reported in one report. Therefore, the number of adverse reactions will not always be the same as the number of individual cases. Also, inadequate information or a lack of additional information may affect the precision of some reported ICSRs, particularly those that deal with medical conditions or medications administered concurrently or concomitantly. For that reason, it is important to interpret the data presented in this study as a means to identify the risk of ADR reporting, rather than to provide a quantification of the risk [54].

## 6. Conclusions

In the present study, according to data extracted and analyzed from the EV database, ceftriaxone (with an average of 40.5 reports per year), ciprofloxacin (an average of 32.0 reports per year), piperacillin/tazobactam (an average of 25.3 reports per year), and meropenem (an average of 13.6 reports per year) had a high incidence of adverse reactions associated with CDI. Moreover, an upward trend in CDI related to some of the most frequently used antibiotics in ICUs was forecasted. In this context, the regulations established by authorities to reduce the consequences of improper and excessive use of antibiotics are very adequate. There is a pressing need to promote responsible antibiotic usage to prevent adverse events and preserve the efficacy of these valuable medications. An increasing rate of severe forms of CDI imposes the necessity to carry out surveillance and monitoring programs for the consumption of antibiotics. Implementing standardized laboratory tests to characterize CDI’s nature accurately is also essential. Collaboration between specialists and strict adherence to antibiotic stewardship programs, hygiene practices, and isolation protocols would significantly contribute to preventing this infection. Similar to other studies, our results suggest that more prudent use of antibiotics such as CFT, CPX, PIP/TAZ, and MER is recommended. Also, retrospective and prospective single- or multicenter cohort trials are needed to advance research in this area. Further studies will offer the opportunity to comprehensively investigate the real risk of antibiotic-associated colitis, providing valuable insights for future signal detection in pharmacovigilance.

## Figures and Tables

**Table 1:** Statistical data referring to the evolution of reports related to *Clostridioides difficile* infection between 1 January 2003 and 31 December 2022.

	CFT	CPX	COL	GEN	LIN	MER	PIP/TAZ
Minimum	2	1	0	0	0	1	1
Maximum	109	86	5	11	7	36	81
Total ICSR	729	640	20	83	54	244	455
Mean	40.5	32.0	1.5	4.9	2.8	13.6	25.3
Standard Deviation	26.0	22.8	1.3	3.1	0.5	12.0	5.5
Confidence Level (95.0%)	12.91852	10.68856	0.8037861	1.573029	1.00671	5.989898	11.63973
lower bound	27.6	21.3	0.7	3.3	1.8	7.6	13.6
upper bound	53.4	42.7	2.3	6.5	3.8	19.5	36.9

CFT—ceftriaxone; COL—colistimethate; CPX—ciprofloxacin; GEN—gentamicin; LIN—linezolid; MER—meropenem; PIP/TAZ—piperacillin and tazobactam.

**Table 2:** Forecasting the cases of *Clostridioides dificille* infection for 2023–2025.

	Intercept	Slope			2023	2024	2025
COL	0.5	0.148352	Predicted values		2.6	2.7	2.9
			95% Prediction Limits	Lower	0	0	0
				Upper	5.8	6.0	6.3
			95% Confidence Limits	Lower	1.0	0.9	0.9
				Upper	4.2	4.5	4.9
			*p*-value		0.1381		
CFT	7.15686	3.5098	Predicted values		73.8	77.4	80.9
			95% Prediction Limits	Lower	30.0	32.8	35.5
				Upper	117.7	121.9	126.2
			95% Confidence Limits	Lower	54.5	56.4	58.3
				Upper	93.2	98.3	103.4
			*p*-value		<0.001		
CPX	6.01053	2.475193	Predicted values		58.0	60.5	62.9
			95% Prediction Limits	Lower	16.3	18.2	20.1
				Upper	99.7	102.7	105.8
			95% Confidence Limits	Lower	40.4	41.6	42.7
				Upper	75.6	79.3	83.1
			*p*-value		<0.05		
GEN	1.77206	0.345588	Predicted values		8.0	8.3	8.7
			95% Prediction Limits	Lower	1.8	2.0	2.2
				Upper	14.2	14.7	15.1
			95% Confidence Limits	Lower	5.2	5.3	5.4
				Upper	10.8	11.4	12.0
			*p*-value		<0.05		
LIN	0.122807	0.27193	Predicted values		5.6	5.8	6.1
			95% Prediction Limits	Lower	2.1	2.4	2.6
				Upper	9.0	9.3	9.6
			95% Confidence Limits	Lower	4.1	4.2	4.4
				Upper	7.0	7.4	7.8
			*p*-value		<0.001		
MER	−6.05229	2.06398	Predicted values		33.2	35.2	37.3
			95% Prediction Limits	Lower	21.3	23.2	25.0
				Upper	45.0	47.3	49.5
			95% Confidence Limits	Lower	27.9	29.6	31.2
				Upper	38.4	40.9	43.4
			*p*-value		<0.0001		
PIP/TAZ	−9.65359	3.67699	Predicted values		60.2	63.9	67.6
			95% Prediction Limits	Lower	29.2	32.3	35.5
				Upper	91.3	95.4	99.7
			95% Confidence Limits	Lower	46.5	49.1	51.6
				Upper	73.9	78.7	83.5
			*p*-value		<0.0001		

CFT—ceftriaxone; COL—colistimethate; CPX—ciprofloxacin; GEN—gentamicin; LIN—linezolid; MER—meropenem; PIP/TAZ—piperacillin and tazobactam. Blue color was used for predicted values.

## Data Availability

Data are contained within the article.

## Abbreviations

ADRs	Adverse drug reactions
AI	Number of infections/year
AMI	Amikacin
CD	*Clostridioides difficile*
CDI	*Clostridioides difficile* infection
CEF	Ceftazidime
CFT	Ceftriaxone
CI	Confidence interval
CLI	Clindamycin
COL	Colistimethate/Colistin
CPX	Ciprofloxacin
EMA	European Medicines Agency
EV	EudraVigilance
FAERS	Food and Drug Administration Adverse Event Reporting System
GEN	Gentamicin
ICSR	Individual Case Safety Report
ICU	Intensive Care Unit
IM	Average index of modification
IMI	Imipenem/Cilastatin
LEV	Levofloxacin
LIN	Linezolid
MER	Meropenem
PIP/TAZ	Piperacilin/Tazobactam
ROR	Reporting odds ratio
SE	Standard error
USD	United States Dollars
